# Estradiol regulates osteoclast sialylation via ST3Gal1 in postmenopausal osteoporosis

**DOI:** 10.1038/s41413-025-00498-x

**Published:** 2026-02-12

**Authors:** Ce Dou, Yang Dan, Ziyang Zhang, Xialin Li, Ying Qu, Yutong Wu, Zhongrong Zhang, Shuquan Guo, Jianzhong Xu, Fei Luo

**Affiliations:** 1 Department of Orthopedics, Southwest Hospital, Army Medical University, Chongqing, China; 2 Department of Orthopedics, Union Hospital, Tongji Medical College, Huazhong University of Science and Technology, Wuhan, China; 3 Department of Orthopedics, Shenzhen NanShan People’s Hospital, Shenzhen, China; 4 Department of Orthopedics of Jiangbei Campus, The First Affiliated Hospital of Army Medical University, Chongqing, China; 5 Department of Orthopaedics, The First Affiliated Hospital of Chongqing Medical University, Chongqing, China

**Keywords:** Endocrine system and metabolic diseases, Bone

## Abstract

Estrogen deficiency after menopause accelerates bone loss by stimulating osteoclast formation and activity, but the molecular pathways that link estrogen signaling to osteoclast regulation remain incompletely defined. Here, we identify the sialyltransferase ST3GAL-I as a key mediator of RANKL-induced osteoclastogenesis. RANKL activates c-FOS to drive ST3GAL1 transcription, whereas estrogen-bound ERα competes with TRAF6 and suppresses this c-FOS–dependent induction. In a clinical cohort of pre-menopausal and post-menopausal women with or without osteoporosis, serum total and α-2,3-linked sialic acid levels increased with age and were highest in post-menopausal osteoporotic patients. Single-cell RNA sequencing of human bone revealed that osteoclasts form a prominent cluster only after menopause, where FOS, CTSK, and ST3GAL1 are strongly co-expressed, and the estrogen-responsive gene PGR is down-regulated. Additionally, in vivo experiments showed that sialidase treatment in estrogen-deficient models effectively reduced osteoclast-mediated bone loss, mimicking the effects of estradiol. These findings define a direct molecular link between loss of estrogen and activation of a FOS–ST3GAL1 sialylation pathway in osteoclasts, providing mechanistic insight into the enhanced bone resorption characteristic of post-menopausal osteoporosis.

## Introduction

Osteoporosis is often asymptomatic in its early stages, as the reduction in bone mineral density typically does not directly impair quality of life. However, osteoporosis-associated fractures significantly diminish health-related quality of life, particularly in older adults.^1^ Post-menopausal osteoporosis, the most prevalent form of osteoporosis, results from decreased estrogen levels following menopause. This estrogen deficiency triggers a rapid phase of bone loss, predominantly in trabecular bone, with an annual rate of 3%–5% over the first 5–10 years.^2^ The risk of osteoporotic fractures escalates with age, with women experiencing nearly double the fracture incidence of men.^3^ Bone loss in post-menopausal women occurs in two stages: an initial rapid phase lasting 3–5 years, primarily affecting trabecular bone, followed by a slower, long-term loss of both cortical and trabecular bone over the next 10–20 years.^4,5^ A hallmark of post-menopausal osteoporosis is the dysregulation of bone remodeling, where bone resorption consistently exceeds bone formation.^6^ At the cellular level, bone remodeling is accomplished by two specialized cells: bone-forming osteoblasts and bone-resorbing osteoclasts. Estrogen receptors (ERs) have been detected in osteoblasts and osteoclasts, including ERα and ERβ isoforms, which bind to different ligands, mediate different effects, and have different spatial distributions.^5^

Osteoclasts, the primary bone-resorbing cells, are large multinucleated cells derived from myeloid macrophages and monocytes. Their differentiation and activation depend on two essential cytokines: macrophage colony-stimulating factor (M-CSF) and receptor activator of NF-κB ligand (RANKL).^7,8^ Estrogen plays a critical role in regulating bone resorption by modulating the RANKL-RANK-osteoprotegerin (OPG) axis, acting as an anti-resorptive hormone to limit osteoclast activity and bone loss.^9^ In the absence of estrogen, such as in post-menopausal women, elevated RANKL expression enhances osteoclast differentiation and function.^10,11^ Inflammatory cytokines such as TNF, IL-1, and IL-6, secreted by mesenchymal stem cells (MSCs) and lymphocytes, further promote osteoclastogenesis.^10^ Beyond these indirect pathways, estrogen also directly inhibits osteoclast differentiation by disrupting RANK signaling^12^ and induces osteoclast apoptosis at low concentrations (0.01 nmol/L).^13^ In menstruating women, serum estradiol levels range from 0.15 nmol/L to 3 nmol/L, but these levels drop below 0.1 nmol/L after menopause.^14^ Despite these insights, important observations remain unexplained. Osteoclast numbers increase markedly in post-menopausal women despite residual circulating estradiol, whereas osteoclast function is preserved during pregnancy when estradiol levels can surge to 70 nmol/L. These findings suggest that the regulatory effects of estrogen on osteoclasts are context-dependent and likely involve additional modulatory mechanisms beyond simple hormone concentration.

Sialylation, mediated by sialyltransferases (STs), is the enzymatic process that transfers sialic acids (SAs) to glycoconjugates in various linkages. SAs are a family of nine-carbon acidic monosaccharides, commonly found on both N- and O-linked glycans, attached to galactose (Gal) or N-acetylgalactosamine (GalNAc) through α2,3- or α2,6-linkages.^15^ Sialylated glycoconjugates play pivotal roles in several biological processes, including cell adhesion, hematopoietic differentiation, and virus-cell interactions.^16–18^ SA-binding immunoglobulin-type lectins (Siglecs), which are primarily expressed on immune cells, specifically recognize these sialylated structures. Recent research has shown a positive correlation between total serum sialic acid levels and age in post-menopausal women.^19^ In contrast, men exhibit an age-related decline in serum SA levels, suggesting a potential regulatory role of estrogen in sialylation. Furthermore, sialylation of cell surface glycoconjugates has been identified as critical for osteoclastogenesis.^20^ Despite these findings, the specific impact of estrogen on SA metabolism and sialylation remains poorly understood.

In this study, we investigated how estrogen regulates osteoclast formation and function. We identify the sialyltransferase ST3GAL-I as a key mediator of RANKL-induced osteoclastogenesis: RANKL activates c-FOS to drive ST3Gal1 transcription, whereas estrogen-bound ERα competes with TRAF6 and suppresses this pathway. In a patient cohort, serum total and α-2,3-linked sialic acid levels increased with age and were highest in post-menopausal women with osteoporosis. Single-cell RNA-seq of human bone showed that osteoclasts appear as a prominent cluster only after menopause, with strong co-expression of FOS, CTSK, and ST3GAL1 and reduced expression of the estrogen-responsive gene PGR. Together, these findings link loss of estrogen to activation of a FOS–ST3GAL1 sialylation program in osteoclasts and to the heightened bone resorption of post-menopausal osteoporosis.

## Results

### RANKL-driven ST3Gal1 upregulation is crucial for osteoclast lineage commitment and fusion

**Fig. 1:**
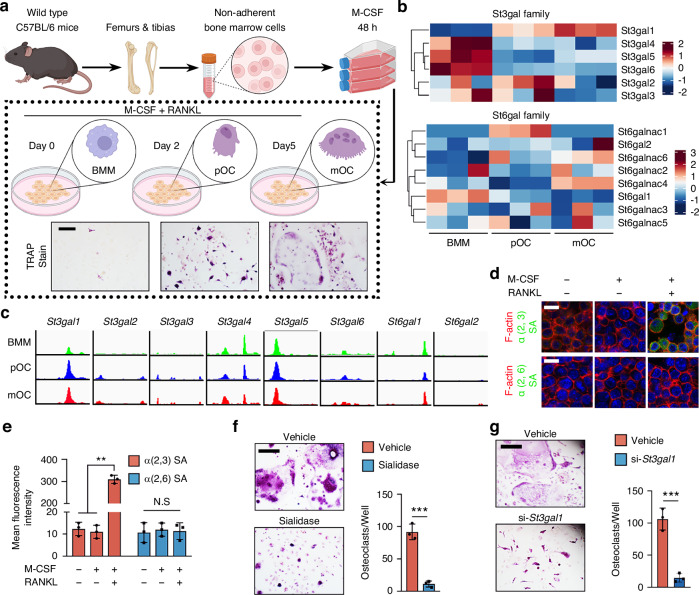
RANKL induces St3gal1 expression is required for osteoclast formation. **a** Schematic of the differentiation process of bone marrow macrophages (BMMs) from wild-type C57BL/6 mice into pre-osteoclasts (pOC) and mature osteoclasts (mOC) under M-CSF and RANKL stimulation over a 5-day period. TRAP staining shows osteoclast formation at different stages. Scale bar = 100 μm. **b** Heatmap showing the expression levels of the mouse sialyltransferase (ST) gene family during osteoclast differentiation from BMMs to pOCs and mOCs. **c** ATAC-seq data illustrating chromatin accessibility changes at the loci of ST3Gal1 and ST6Gal family members during osteoclast differentiation. **d** Immunofluorescence staining of α2,3 and α2,6 sialylation on BMMs after treatment with M-CSF and/or RANKL. Scale bar = 20 μm. **e** Quantification of mean fluorescence intensity of α2,3 and α2,6 sialic acids on BMMs treated with M-CSF and RANKL (*n* = 3). **f** TRAP staining of osteoclast cultures treated with vehicle or sialidase. Scale bar = 100 μm. Quantification of the number of multinucleated osteoclasts per well in vehicle- and sialidase-treated cells (*n* = 3). **g** TRAP staining of osteoclasts treated with vehicle or siRNA targeting ST3Gal1 (si-ST3Gal1). Scale bar = 100 μm. Quantification of the number of multinucleated osteoclasts per well in vehicle- and si-ST3Gal1-treated cells (*n* = 3). Data are presented as mean ± SD; statistical significance was determined by one-way ANOVA, with *P < 0.05, **P < 0.01, ***P < 0.001

We began by isolating bone marrow cells from the hind limbs of C57BL/6 mice and stimulating them with M-CSF (50 ng/mL) for 48 h to generate bone marrow macrophages (BMMs). Following this, BMMs were exposed to RANKL to induce the formation of tartrate-resistant acid phosphatase-positive (TRAP^+^) macrophages, which differentiated into pre-osteoclasts (pOCs) and mature osteoclasts (mOCs) (Fig. 1a). To identify sialyltransferases involved in osteoclastogenesis, we performed RNA sequencing (RNA-seq) on BMMs, pOCs, and mOCs. Clustering of the sialyltransferase (ST) gene family revealed that ST3Gal1, which encodes the enzyme responsible for α2,3 sialylation, was significantly upregulated during osteoclastogenesis, while ST6Gal1, responsible for α2,6 sialylation, was down-regulated (Fig. 1b). Western blot analysis further confirmed the upregulation of ST3Gal1 at the protein level (Fig. S1). ATAC-seq profiling of BMM, pOC, and mOC results are consistent with bulk RNA-seq, showing the increased chromatin accessibility of ST3Gal1 during osteoclastogenesis (Fig. 1c). We next analyzed surface α2,3 and α2,6 sialylation during osteoclast differentiation (Fig. 1d). RANKL—but not M-CSF—induced robust α2,3 sialylation, with no significant changes observed in α2,6 sialylation (Fig. 1e). To assess the functional role of sialylation, we treated BMMs with sialidase to remove surface sialic acids during RANKL-induced osteoclastogenesis and performed TRAP staining. While sialidase treatment did not affect TRAP^+^ pOC formation, it significantly impaired the fusion of pOCs into multinucleated mOCs (Fig. 1f). Additionally, ST3Gal1 knockdown using siRNA mimicked the effects of sialidase, strongly inhibiting multinucleated osteoclast formation (Fig. 1g).

### RANKL activates *ST3Gal1* transcription through c-FOS binding to the *ST3Gal1* enhancer

**Fig. 2:**
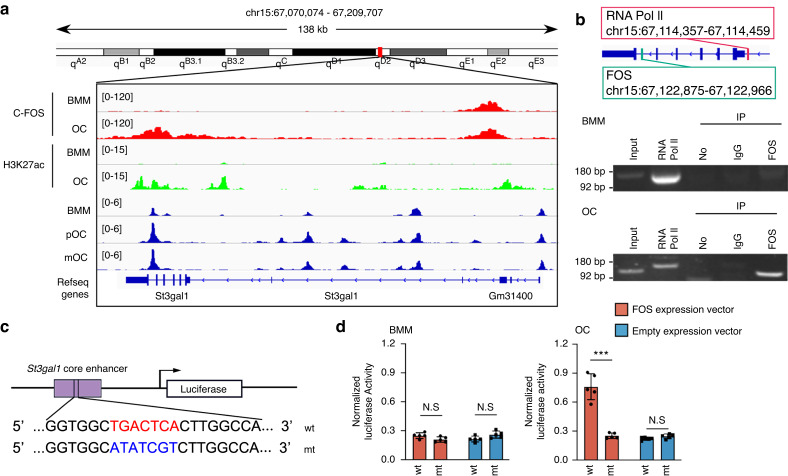
RANKL activates St3gal1 transcription in a c-FOS-dependent way. **a** Genome browser snapshot displaying a 138 kb region surrounding the ST3Gal1 gene locus, highlighting its core enhancer. ChIP-seq data reveal a prominent FOS binding peak, indicating the region bound by the FOS transcription factor, with additional H3K27ac ChIP-seq tracks comparing BMMs and OCs to show enhanced histone acetylation in OCs, alongside ATAC-seq data demonstrating increased chromatin accessibility at the c-FOS binding site in response to RANKL stimulation. **b** Chromatin immunoprecipitation (ChIP) followed by PCR confirming the binding of FOS to the ST3Gal1 core enhancer across conditions with and without RANKL treatment, Input DNA was included as a control; RNA polymerase II(Pol II) served as a positive control; Immunoglobulin G (IgG) and no antibody served as negative controls. **c** Schematic representation of the wild-type (wt) and mutant (mt) ST3Gal1 core enhancer sequences. The FOS binding site (TGACTCA) in the wild-type enhancer is mutated (ATATCGT) in the mutant construct. Both constructs were cloned upstream of a luciferase reporter. **d** Luciferase reporter assay showing normalized luciferase activity in BMMs and OCs transfected with the wild-type or mutant ST3Gal1 enhancer and co-transfected with either a FOS expression vector or an empty vector (*n* = 5). Data are presented as mean ± SD; statistical significance was determined by one-way ANOVA, with *P < 0.05, **P < 0.01, ***P < 0.001, N.S. not significant

To investigate the transcriptional regulation of ST3Gal1 during osteoclastogenesis, we first analyzed the promoter region of ST3Gal1 and identified potential binding sites for c-FOS, a critical transcription factor downstream of RANKL signaling. Chromatin immunoprecipitation (ChIP)-PCR assays and ChIP-seq data were used to determine whether c-FOS directly interacts with these regulatory regions of ST3Gal1. The ChIP-seq results showed enrichment of c-FOS, H3K27ac at the ST3Gal1 locus, particularly around the core enhancer region, following RANKL stimulation in BMMs relative to OCs (Fig. 2a). ChIP-PCR further confirmed that c-FOS binds to the core enhancer of the ST3Gal1 promoter, with RNA Pol II also showing increased binding, indicating active transcription (Fig. 2b). To assess the functional relevance of this interaction, we generated a reporter construct containing the wild-type ST3Gal1 enhancer region, as well as a mutant construct in which the c-FOS binding site (TGACTCA) was altered (Fig. 2c). Transcriptional activity was measured in cells transfected with these constructs. In cells expressing the wild-type enhancer, RANKL stimulation resulted in a significant increase in transcriptional activity relative to cells without RANKL treatment. However, mutation of the c-FOS binding site abrogated this response, demonstrating that the TGACTCA sequence is essential for c-FOS-dependent transcription of ST3Gal1 (Fig. 2d). These results establish that RANKL-induced activation of ST3Gal1 transcription is mediated by c-FOS binding to its promoter region. The c-FOS-ST3Gal1 axis is therefore a critical component of the molecular pathway through which RANKL promotes osteoclast differentiation.

### ST3Gal1 expression and α2,3 sialylation are increased in ovariectomized mice and are dependent on RANKL signaling

**Fig. 3:**
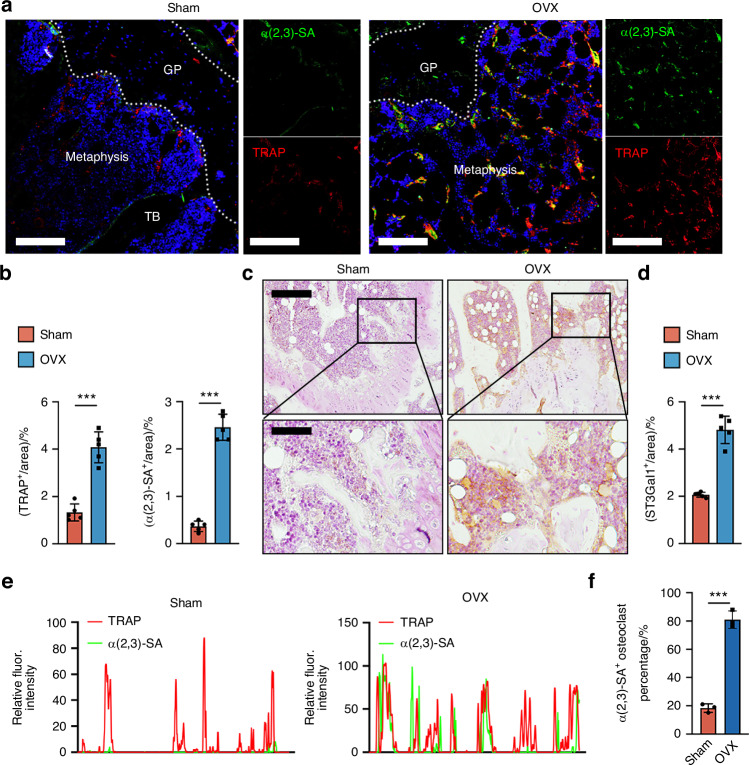
Bone sialylation level and ST3Gal1 expression are increased in ovariectomized mouse. **a** Representative immunofluorescent images of femur sections from sham and OVX mice showing α2,3-sialylation (green) and TRAP^+^ osteoclasts (red). The growth plate (GP), metaphysis, and trabecular bone (TB) regions are indicated. Scale bars = 100 μm. **b** Quantification of TRAP^+^ area and α2,3-sialic acid (SA)^+^ area as a percentage of the total bone area in sham and OVX mice (*n* = 5). **c** Representative immunohistochemical (IHC) images of ST3Gal1 expression in femur sections from sham and OVX mice. Insets show magnified views of the indicated regions. Scale bars = 200 μm (top panels), 100 μm (bottom panels). **d** Quantification of ST3Gal1^+^ area as a percentage of the total bone area in femur sections from sham and OVX mice (*n* = 5). **e** Line plot of relative fluorescence intensity profiles of TRAP and α(2,3)-SA signals along selected regions in (**a**), demonstrating colocalization. **f** Quantification of α(2,3)-SA⁺ osteoclasts (TRAP⁺) as a percentage of total osteoclasts in sham and OVX mice (*n* = 3). Data are presented as mean ± SD; statistical significance was determined by one-way ANOVA, with *P < 0.05, **P < 0.01, ***P < 0.001

To explore the role of ST3Gal1 and α2,3 sialylation in the context of post-menopausal osteoporosis, we utilized an ovariectomized (OVX) murine model. Co-immunofluorescent staining of α2,3 SA and TRAP was performed on distal femur sections from both sham-operated and OVX mice (Fig. 3a). The analysis revealed a significant increase in α2,3 sialylation in the trabecular bone of OVX mice compared to controls, particularly in TRAP^+^ osteoclasts (Fig. 3b). This suggests a potential link between enhanced α2,3 sialylation and increased osteoclast activity in estrogen-deficient conditions. We further assessed the expression of ST3Gal1 using immunohistochemistry (IHC) on distal femur sections. Consistent with the increase in α2,3 sialylation, ST3Gal1 expression was markedly upregulated in the OVX group relative to sham-operated mice (Fig. 3c, d). To quantify the α2,3-SA-positive cells among osteoclasts, we analyzed the co-localized signals of α2,3-SA and TRAP (Fig. 3e), showing that α2,3-SA^+^ osteoclast number is much higher in OVX mice (Fig. 3f). These findings indicate that the upregulation of ST3Gal1 contributes to enhanced osteoclast activity and bone resorption in the OVX model. To investigate whether RANKL signaling is necessary for this upregulation in vivo, we used a conditional RANKL knockout model by crossing *RANKL*^*fl*/fl^ mice with Dmp1-Cre to generate *RANKL*^ΔDmp1^ mice, where RANKL is specifically deleted in osteocytes. In these RANKL-deficient mice, both ST3Gal1 expression and α2,3 sialylation levels were significantly reduced compared to wild-type controls (Fig. S2). This confirms that RANKL is a critical driver of ST3Gal1-mediated sialylation in osteoclasts during bone remodeling.

### Estradiol inhibits RANKL-induced ST3Gal1 transcription by competing with TRAF6 and suppressing c-FOS activity

**Fig. 4:**
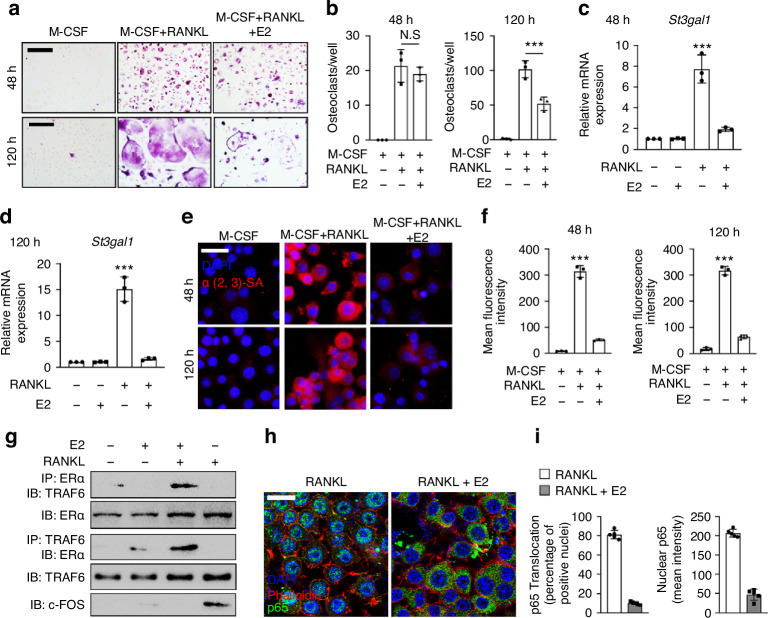
Estradiol inhibits RANKL-activated St3gal1 transcription via competitively binding with TRAF6, reducing c-Fos activity. **a** TRAP staining of osteoclasts cultured with M-CSF, RANKL, or RANKL + estradiol (E2) for 48 and 120 h. Scale bars = 100 μm. **b** Quantification of osteoclasts per well in the conditions described in (**a**) at 48 and 120 h. qPCR analysis of St3gal1 mRNA levels in osteoclasts treated with M-CSF, RANKL, or RANKL + E2 for 48 h (**c**) and 120 h (**d**). **e** Immunofluorescent staining of α2,3 sialylation (red) in osteoclasts treated with M-CSF, RANKL, or RANKL + E2 at 48 and 120 h. DAPI (blue) is used to counterstain nuclei. Scale bar = 20 μm. **f** Quantification of mean fluorescence intensity of α2,3 sialic acid staining in osteoclasts treated as described in (**e**). **g** Co-immunoprecipitation (IP) of ERα and TRAF6 in osteoclasts treated with RANKL or RANKL + E2, followed by immunoblotting (IB) to detect TRAF6, ERα, and c-FOS. **h** Immunofluorescence analysis of p65 nuclear translocation in osteoclasts treated with RANKL or RANKL + E2. DAPI (blue) counterstains nuclei, and Phalloidin (green) stains actin. Scale bar = 20 μm. **i** Quantification of p65 nuclear translocation (percent of positive nuclei and mean intensity) in osteoclasts treated with RANKL or RANKL + E2 (*n* = 5). Data are presented as mean ± SD; statistical significance was determined by one-way ANOVA, with *P < 0.05, **P < 0.01, ***P < 0.001

To investigate how estrogen modulates ST3Gal1-mediated α2,3 sialylation during osteoclastogenesis, we first examined the effect of 17β-estradiol (E2) on pOC and mOC formation. While E2 pretreatment did not influence pOC differentiation, it significantly reduced the formation of multinucleated mOCs after 120 h of RANKL stimulation (Fig. 4a, b). Quantitative PCR (qPCR) analysis confirmed that E2 markedly suppressed RANKL-induced ST3Gal1 transcription at both the 48-h and 120-h time points (Figs. 4c, d and S4). Immunostaining of α2,3 sialylation on the osteoclast surface demonstrated that E2 effectively reduced ST3Gal1-mediated α2,3 sialylation in both pOCs and mOCs (Fig. 4e, f), suggesting that estrogen exerts a broad inhibitory effect on osteoclast sialylation. To elucidate the molecular mechanism, we explored whether estrogen impacts RANKL signaling via the ERα. Co-immunoprecipitation (IP) experiments revealed that ERα binds with TRAF6 in response to RANKL stimulation, indicating that ERα competes with TRAF6 for interaction with downstream signaling components (Fig. 4g). This interaction inhibited the activation of c-FOS, a key transcription factor required for ST3Gal1 expression, as shown by reduced c-FOS levels in E2-treated cells (Fig. 4g). Additionally, we examined the effect of E2 on NF-κB signaling, a pathway downstream of c-FOS activation. Immunofluorescence and confocal analysis of the p65 subunit demonstrated that RANKL-induced nuclear translocation of p65 was attenuated by E2 treatment, suggesting a broader suppression of osteoclastogenic signaling (Fig. 4h, i). These results highlight a multi-level inhibitory effect of estrogen on osteoclastogenesis, mediated through suppression of c-FOS activity and downstream signaling pathways.

### Sialidase treatment effectively reverses osteoclast-induced bone loss and reduces α2,3 sialylation in ovariectomized mice

**Fig. 5:**
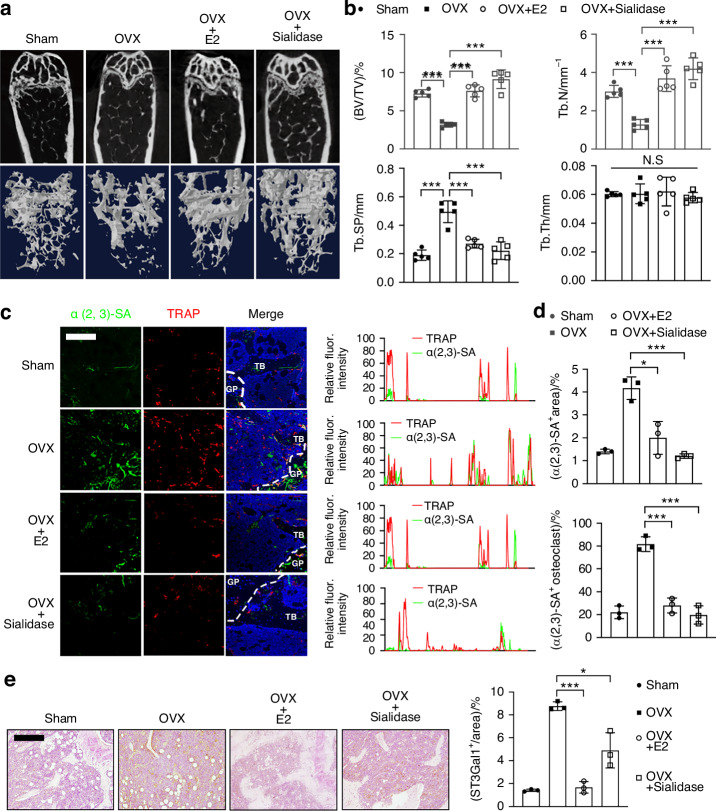
Sialidase treatment restored trabecular bone loss in OVX mice. **a** Representative micro-CT images of distal femoral trabecular bone in four groups of mice: Sham-operated control (Sham), ovariectomized (OVX), OVX treated with estrogen (OVX + E2), and OVX treated with sialidase (OVX + Sialidase). **b** Quantitative analysis of bone parameters from the micro-CT data: Bone volume/total volume (BV/TV), trabecular number (Tb.N), trabecular separation (Tb.Sp), and trabecular thickness (Tb.Th) (*n* = 5). **c** Representative immunofluorescence staining of trabecular bone showing α(2,3)-linked sialic acid (α(2,3)-SA, green), tartrate-resistant acid phosphatase (TRAP, red), and DAPI (blue). Right panels show fluorescence intensity profiles and quantification of α(2,3)-SA⁺ area and α(2,3)-SA⁺ TRAP⁺ osteoclasts in different groups. **d** Quantification of α(2,3)-SA⁺ area and percentage of α(2,3)-SA⁺ TRAP⁺ osteoclasts (*n* = 3). **e** Representative IHC staining of ST3Gal1 in distal femur sections from each group, with quantification of ST3Gal1⁺ area shown on the right (*n* = 3). Data are presented as mean ± SD; statistical significance was determined by one-way ANOVA, with *P < 0.05, **P < 0.01, ***P < 0.001

Building upon our previous findings that α2,3 sialylation mediated by ST3Gal1 is elevated in the trabecular bone of ovariectomized (OVX) mice, we evaluated whether removing α2,3 sialic acids could mitigate bone loss caused by estrogen deficiency. For this purpose, we administered sialidase, an enzyme that cleaves α2,3 sialic acids, systemically to OVX mice for 4 weeks. The effects of sialidase were compared with those of estrogen replacement therapy (E2), and both groups were assessed for changes in bone architecture using micro-CT (μCT) (Figs. 5a and S5). Our results show that sialidase treatment significantly improved key bone parameters, including increased trabecular bone volume (BV/TV) and trabecular number (Tb.N), as well as decreased trabecular separation (Tb.Sp), effectively mimicking the beneficial effects of E2 treatment (Fig. 5b). These improvements indicate a reduction in osteoclast-induced bone resorption. Histological analysis corroborated these findings, with TRAP staining showing a significant decrease in the number and size of multinucleated osteoclasts in the sialidase-treated group compared to untreated OVX controls (Fig. S3). Furthermore, co-immunostaining of α2,3-sialylated TRAP^+^ osteoclasts in the femur revealed that both E2 and sialidase treatments reduced the proportion of α2,3-SA, TRAP double-positive cells in OVX mice (Fig. 5c, d). Immunohistochemical analysis confirmed a similar reduction in ST3GAL-I expression levels in the bone tissue of treated mice (Fig. 5e). These results demonstrate that targeting excessive α2,3 sialylation with sialidase can reverse bone loss and osteoclast activity in estrogen-deficient conditions, offering a potential therapeutic strategy akin to hormone replacement therapy.

### Elevated serum α-2,3 sialic acid levels and single-cell transcriptomics link estrogen deficiency to osteoclast-specific sialylation

**Fig. 6:**
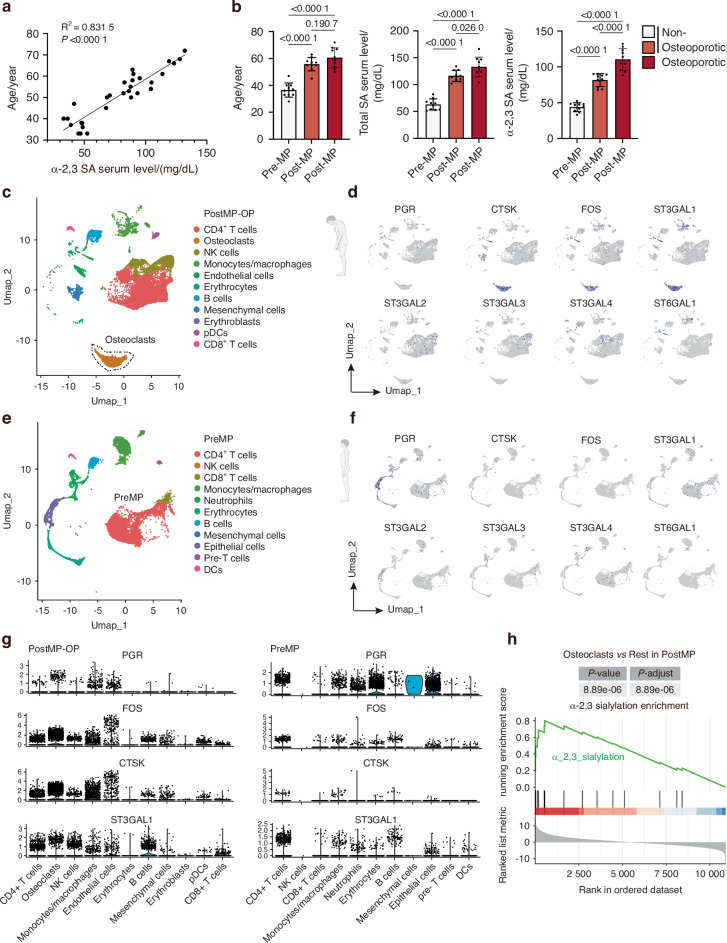
Loss of estrogen after menopause increases serum α-2,3 sialylation and activates a sialylation program in osteoclasts. **a** Correlation of serum α-2,3 sialic acid (SA) concentration with age (*n* = 30); simple linear regression with coefficient of determination (R^2^) and p-value indicated. **b** Age distribution (left), total serum SA (middle), and α-2,3 SA (right) in pre-menopausal (Pre-MP), post-menopausal non-osteoporotic (Post-MP), and post-menopausal osteoporotic (Post-MP-OP) women; bars show mean ± SD and p values from Welch’s t-test. **c** UMAP of single-cell transcriptomes from Post-MP-OP bone samples colored by annotated cell types; the osteoclast cluster is outlined. **d** UMAP feature plots showing expression of estrogen-responsive genes (PGR, FOS), osteoclast markers (CTSK) and sialyltransferases (ST3GAL1–4, ST6GAL1) in Post-MP-OP cells. **e** UMAP of Pre-MP bone samples with cell-type annotations. **f** Corresponding feature plots of PGR, FOS, CTSK, and ST3GAL1–4, ST6GAL1 in Pre-MP cells. **g** Violin plots comparing cell-type-specific expression of PGR, FOS, CTSK, and ST3GAL1 in Post-MP-OP (left) and Pre-MP (right) samples. **h** Gene set enrichment analysis (GSEA) of the α-2,3 sialylation gene set in Post-MP-OP osteoclasts versus all other Post-MP-OP cell types; *P*-value, and FDR (adjusted *P*-value) are indicated

To examine the relationship between systemic sialylation and post-menopausal bone loss, we measured serum α-2,3 sialic acid (α-2,3 SA) levels in an expanded clinical cohort that included pre-menopausal women and post-menopausal subjects with or without osteoporosis. Serum α-2,3 SA concentration showed a strong positive correlation with age (Fig. 6a), and was significantly higher in post-menopausal osteoporotic patients than in either pre-menopausal or age-matched post-menopausal non-osteoporotic controls (Fig. 6b). To identify the cellular sources of this signal, we performed scRNA-seq on trabecular bone and bone-marrow cells from four pre-menopausal (preMP) and four post-menopausal osteoporotic (postMP-OP) donors. UMAP visualization revealed all expected marrow and stromal cell types (Fig. 6c, e). A prominent osteoclast cluster was evident only in the postMP-OP samples, whereas osteoclasts were not detected as a distinct population in the preMP cohort. Consistent with diminished estrogen signaling after menopause, the classical estrogen-responsive gene PGR was strongly expressed in preMP cells but markedly reduced in postMP-OP cells (Figs. 6d, f, g and S8). Within postMP-OP osteoclasts, among the sialyltransferases, ST3GAL1 was specifically highly expressed alongside the osteoclast markers CTSK and ACP5, and showed coordinated induction of the transcription factor FOS (Fig. 6d, g). Gene Set Enrichment Analysis (GSEA) using a “α-2,3 sialylation” gene set demonstrated significant enrichment of this pathway specifically in postMP-OP osteoclasts compared with all other cell types (Fig. 6h), directly linking the clinical rise in serum α-2,3 SA to cell-type–specific upregulation of sialylation machinery. The trending of ST3Gal1 expression is similar in mouse scRNA-seq data (Figs. S6 and S7). Complementary pathway analyses reinforced these findings: GO terms upregulated in postMP-OP samples were enriched for “glycosylation” and “protein glycosylation,” whereas down-regulated terms included “estrogen receptor signaling pathway.” KEGG analysis highlighted “osteoclast differentiation” among the top upregulated pathways (Fig. S9). Together, these results demonstrate that loss of estrogen after menopause is accompanied by increased α-2,3 sialylation and activation of a FOS–CTSK–ST3GAL1 program within osteoclasts, providing a mechanistic link between systemic sialylation and osteoclast-driven bone resorption in post-menopausal osteoporosis.

## Discussion

In this study, we identified ST3Gal1 as a key regulator of osteoclast differentiation and function in post-menopausal osteoporosis. RANKL-induced ST3Gal1 upregulates α2,3-sialylation, which is essential for osteoclast fusion and bone resorption. Clinical data from post-menopausal women revealed that elevated serum α2,3-sialic acid levels correlate with osteoporosis severity, and scRNA-seq analysis confirmed ST3Gal1 enrichment in osteoclasts. Estradiol inhibits this pathway by disrupting the TRAF6-c-FOS axis, suppressing ST3Gal1 expression and osteoclast activity. In vivo, estrogen deficiency in ovariectomized mice increased ST3Gal1 expression and sialylation, enhancing bone resorption. Sialidase treatment reduced bone loss, suggesting that targeting sialylation is a potential therapeutic strategy for osteoporosis.

The interaction between estradiol and sialylation is particularly relevant in understanding post-menopausal osteoporosis. With declining estradiol levels during menopause, there is a corresponding increase in sialic acid levels, which may exacerbate bone loss by promoting osteoclast activity. Studies have indicated that estrogen replacement therapy can restore both estradiol and sialic acid levels, thereby enhancing bone health.^21,22^ Notably, the inhibition of NF-κB translocation in osteoclasts upon estrogen treatment has been described, suggesting a mechanism by which estradiol may suppress osteoclast activity and reduce bone resorption.^23^ Moreover, specific studies have highlighted the role of selective estrogen receptor modulators (SERMs) that mimic estrogen’s beneficial effects on bones without some adverse effects associated with traditional hormone replacement therapy. These SERMs have been shown to influence glycosylation patterns, including sialylation, suggesting a potential therapeutic avenue for managing osteoporosis.^24,25^ Estrogen treatment has been shown to enhance the sialylation of immunoglobulin G (IgG), which may reduce its pathogenicity in autoimmune conditions like rheumatoid arthritis.^26^ Research has also shown that IgG complexes sialylation can affect osteoclast formation, potentially leading to increased bone loss in autoimmune conditions.^27,28^ This suggests that estradiol’s influence on sialic acid levels may extend beyond direct effects on bone cells to include modulation of immune factors that impact bone health.

Altered sialylation patterns on osteoclast precursors can modulate their differentiation and resorptive activity. It was reported that the desialylation of cell surface glycoproteins on osteoclast precursors impairs their differentiation into mature multinucleated osteoclasts. Furthermore, sialic acids on osteoprotegerin, a decoy receptor for RANKL affecting its stability and interaction with RANKL, thereby influencing osteoclastogenesis.^20,29^ We previously showed that α2,3-sialylation is essential for cell recognition among preosteoclasts in initiating cell fusion.^30^ Apart from our findings in osteoporosis, altered sialylation patterns on glycoproteins could influence the behavior of immune cells involved in ankylosing spondylitis (AS). For instance, changes in sialylation may affect the activation and migration of T cells and natural killer cells, which are implicated in AS pathogenesis.^31,32^ Additionally, sialylation of cytokine receptors may modulate cytokine signaling pathways, influencing inflammatory responses and new bone formation in AS.^32,33^ In rheumatoid arthritis (RA), higher serum levels of Neu5Ac were significantly associated with the presence as well as severity of RA.^34^ A recent study further confirmed that loss of α2-6 sialylation promotes the transformation of synovial fibroblasts into a pro-inflammatory phenotype in RA.^35^

Sialylation is also highly relevant in immunology due to its pivotal role in regulating immune cell interactions, signaling pathways, and responses.^36–38^ Sialic acids at the terminal positions of glycans can modulate the immune system by engaging with Siglecs on immune cells, thereby influencing processes like cell adhesion, migration, and activation.^39–41^ In our scRNA-seq analysis of bone and bone marrow from osteoporotic patients, we found clear differences in both immune-cell composition and the expression of sialylation-related genes within the bone microenvironment. For example, post-menopausal osteoporotic samples showed a relative expansion of monocyte/macrophage populations and reduced expression of the estrogen-responsive gene PGR, together with higher levels of key sialyltransferase ST3GAL1 in osteoclasts. These observations indicate that altered sialylation accompanies the immune-cell shifts and loss of estrogen signaling characteristic of post-menopausal osteoporosis, highlighting a potential association between sialylation changes and the altered bone immune microenvironment.

In conclusion, our study identifies the sialyltransferase ST3Gal1 as a critical mediator of osteoclast function in post-menopausal osteoporosis. We demonstrated that RANKL-induced α2,3-sialylation via ST3Gal1 promotes osteoclast fusion and bone resorption. Estradiol effectively inhibits this process by disrupting the TRAF6-c-FOS axis. Clinical data from post-menopausal women and scRNA-seq analysis confirmed the relevance of ST3Gal1 in osteoclasts. These findings highlight the potential of targeting sialylation as a therapeutic strategy to mitigate osteoclast-driven bone loss in estrogen-deficient conditions.

## Materials and methods

### Mice and treatment

We purchased 3-month-old C57BL/6J (WT, Stock: 000664) mice from Jackson Laboratory (Bar Harbor, ME, USA). The Siglec-15 conditional knockout strain (C57BL/6 background) was obtained from the Mutant Mouse Regional Resource Center (MMRRC) and crossed with LysM-Cre mice from Jackson Laboratory to generate Siglec15ΔLysM mice (whole-body or myeloid-specific knockout), as previously described. Wild-type littermates from Siglec-15 heterozygotes were bred under the same conditions as the knockout mice for controls. Dmp1-Cre and RANKLfl/fl mice were purchased from Jackson Laboratory. Dmp1-Cre heterozygotes were crossed with RANKLfl/fl mice, and offspring were intercrossed to generate WT, Dmp1-Cre, RANKLfl/fl, and RANKLΔDmp1 (conditional Rankl deletion in DMP1^+^ cells) genotypes. Mice were euthanized at 4 or 11 weeks (10–12 per group) using isoflurane overdose for time-course studies. For sialidase injections, 4-week-old mice were anesthetized with ketamine (100 mg/kg, intraperitoneally) and xylazine (10 mg/kg, intraperitoneally). SialEXO 23 α2-3 specific sialidase (5 units, Genovis Inc.) was preincubated in 20 mmol/L Tris (pH 7.5, 37 °C, 1 h) and injected intrafemorally. All protocols were approved by the Army Medical University’s Animal Care and Use Committee.

### RNA sequencing

Total RNA was extracted using the RNeasy Mini Kit (Qiagen, Germany). Paired-end libraries were prepared with the TruSeq™ RNA Sample Preparation Kit (Illumina, USA) following the manufacturer’s protocol. Poly-A mRNA was purified using poly-T oligo-attached magnetic beads, fragmented at 94 °C for 8 min, and converted to cDNA through reverse transcription. Second-strand cDNA synthesis was performed using DNA Polymerase I and RNase H. The cDNA fragments were end-repaired, A-tailed, and ligated to adapters. After purification and PCR enrichment, libraries were quantified with a Qubit® 2.0 Fluorometer (Life Technologies, USA) and validated with an Agilent 2100 Bioanalyzer (Agilent Technologies, USA). Clusters were generated by cBot at 10 pmol/L concentration, and sequencing was conducted on the Illumina NovaSeq 6000 platform.

### ATAC-seq and data analysis

ATAC-seq was performed on approximately 50 000 cells per sample to assess chromatin accessibility. Nuclei were isolated and subjected to tagmentation using Tn5 transposase, followed by PCR amplification to enrich for adapter-ligated fragments. Libraries were sequenced on an Illumina platform, and reads were quality-checked, trimmed, and aligned to the reference genome using Bowtie2. Peaks were called using MACS2, with differential accessibility analyzed via DiffBind. Peaks were annotated to nearby genes with ChIPseeker, and functional enrichment was assessed using GREAT. Data visualization and motif analysis were conducted using R packages and HOMER, respectively.

### Chip-Seq

ChIP-seq experiments were conducted using the BeyoChIP™ Chromatin Immunoprecipitation Assay Kit with Protein A/G Magnetic Beads (P2080S, Beyotime). Sequencing libraries were prepared from 2–10 ng of purified ChIP DNA using the NEBNext Ultra DNA Library Prep Kit for Illumina (NEB) following the manufacturer’s protocol. The completed libraries were quantified using a Qubit fluorometer, Agilent TapeStation 2200, and RT-qPCR with the Kapa Biosystems library quantification kit. Uniquely indexed libraries were pooled in equimolar amounts and sequenced on the Illumina NextSeq500 platform with single-end 75 bp reads at the Dana-Farber Cancer Institute Molecular Biology Core Facilities. The sequencing data have been deposited in the GEO database under accession number GSE151481.

### μCT analysis

Mice were euthanized using isoflurane overdose, followed by perfusion with phosphate-buffered saline (PBS) for 5 min and 10% buffered formalin for another 5 min via the left ventricle. Femurs were dissected and fixed overnight in 70% ethanol, then analyzed using a Bruker μCT Skyscan 1172 system (Kontich, Belgium) at a 10.0 μm isotropic voxel size. Scanning was conducted at 60 kV, 166 μA, and 1 700 ms exposure. 3D reconstructions of the region of interest, including the DBM, were performed using NRecon software (Kontich, Belgium). 3D and 2D analyses were done with CT Analyser (Ver. 1.15.4.0, Kontich, Belgium), evaluating parameters such as Tb.Con, Tb.Sp, BV/TV, Tb.N, and Tb.Th for distal femurs.

### Osteoclast differentiation assay

For TRAP staining, BMMs were seeded in 96-well plates (5 × 10^3^ cells/well) and cultured with M-CSF (50 ng/mL) and RANKL (100 ng/mL). At 0, 24, and 96 h post-stimulation, cells were fixed with 4% paraformaldehyde for 20 min, then stained with TRAP solution (0.1 mg/mL naphthol phosphate, 0.3 mg/mL Fast Red Violet) following the manufacturer’s protocol. TRAP activity was analyzed by colorimetry. For immunofluorescence (IF), BMMs were cultured similarly for 4 days with 1 U/mL SialEXO 23 α2-3 sialidase and 10 μmol/L U0126, then fixed, permeabilized with 0.2% Triton X-100, and blocked. Cells were stained with vinculin antibody (1:500) for 1 h at 37 °C and counterstained with DAPI (1:1 000) for 10 min before fluorescence and confocal microscopy. For the pit formation assay, BMMs were seeded in 96- or 48-well Corning Osteo Assay Surface plates (2 × 10^3^ or 1 × 10^4^ cells/well) and induced with RANKL and M-CSF for 5 days. Cells were removed with bleach solution, and pit formation area was analyzed as previously described.

### Immunohistochemistry, immunofluorescence, and histomorphometry

Femurs were collected, fixed in 4% paraformaldehyde overnight, and decalcified in 10% EDTA (pH 7.4) for 21 days. Samples were dehydrated in 30% sucrose for 24 h and embedded in paraffin or optimal cutting temperature compound (Sakura Finetek). Four-micrometer-thick coronal sections were prepared for hematoxylin and eosin staining. For immunostaining, femurs were fixed for 4 h in 4% paraformaldehyde, decalcified in 0.5 mol/L EDTA at 4 °C for 24 h, and dehydrated in 20% sucrose and 2% polyvinylpyrrolidone (PVP) for 24 h before embedding in 8% gelatin. Forty-micrometer-thick coronal sections were processed for immunostaining using primary antibodies against ST3GAL1 (1:50) and TRAP (1:100) at 4 °C overnight. Immunohistochemical detection was performed with a horseradish peroxidase-streptavidin kit, followed by hematoxylin counterstaining. Sialic acid detection was performed using biotinylated Maackia amurensis lectin II (MAL II) to detect α2,3-linked sialic acids and Sambucus nigra lectin (SNA) to detect α2,6-linked sialic acids, followed by fluorescein-conjugated streptavidin for signal visualization. Imaging was conducted using a Zeiss LSM 780 confocal or Olympus BX51 microscope, and quantitative histomorphometry was performed using OsteoMeasure XP software in a blinded manner.

### ChIP-PCR assay

For the ChIP assay, BMMs were cultured with M-CSF and RANKL for 72 h to detect ST3Gal1 core enhancer DNA binding. Cells were lysed and cross-linked with 1% formaldehyde, followed by DNA fragmentation via micrococcal nuclease (MNase) digestion. Ten percent of the sample was preserved as input, and the remaining supernatant was incubated overnight at 4 °C with ChIP Grade c-FOS antibody (Abcam, ab27793, 10 μg). RNA Polymerase II antibody served as the positive control, and normal Rabbit IgG was the negative control. Immunoprecipitation was performed using ChIP Grade Protein A/G Magnetic Beads (Thermo Fisher Scientific, 26157). Eluted DNA was purified according to the manufacturer’s instructions and prepared for PCR detection.

### Luciferase reporter assay

For the Luciferase reporter assay, wild-type and mutant (TGACTCA to ATATCGT) ST3Gal1 enhancer regions were cloned into pGL3-Basic, co-transfected with pRL-TK into RAW264.7 cells using Lipofectamine 3000, and treated with RANKL (50 ng/mL) for 48 h. Luciferase activity, normalized to Renilla, was then detected.

### Bioinformatics

Gene core enhancer transcription factor binding was predicted using the ENCODE project and ChIP-seq data from the UCSC Genome Browser (https://genome.ucsc.edu/ENCODE). RNA-seq raw and processed data have been deposited in the GEO database under accession number GSE133515.

### Immunoblotting analysis

Cells were lysed in IP buffer (50 mmol/L Tris-HCl, pH 7.5, 150 mmol/L NaCl, 1% Triton X-100, 0.5% sodium deoxycholate) with protease inhibitors. Lysates were immunoprecipitated using primary antibodies against TLR2 (Santa Cruz Biotechnology, sc-21759) and Siglec15 (Thermo Fisher, PA5-48221), followed by Protein A/G absorption (Thermo Fisher, 26149). Immunoprecipitates were separated by SDS-PAGE, transferred to nitrocellulose, and detected using the SuperSignal West Femto Substrate system (Thermo Fisher). For immunoblotting, cells were lysed in IP buffer with 0.5% SDS and incubated overnight at 4°C with primary antibodies against p-ERK, ST3GAL1, c-FOS, ER-alpha, TRAF6, and Siglec15 (1:1 000). Secondary antibody incubation was performed for 1 h (1:2 000).

### Human subjects, serum sialic acid measurement, and scRNA-seq

The study cohort comprised pre-menopausal women (*n* = 10), post-menopausal women without osteoporosis (*n* = 10), and post-menopausal women with osteoporosis (*n* = 10), with serum samples collected from all participants. Total sialic acid levels were quantified using a commercial colorimetric assay, and α-2,3-linked SA levels were measured by a lectin-based ELISA employing Maackia amurensis lectin II (MAL-II), which specifically recognizes α-2,3-linked sialic acids. Bone and bone-marrow specimens were obtained from four post-menopausal osteoporotic (postMP-OP) patients (ages 65–73) undergoing spinal surgery and four pre-menopausal (preMP) women (ages 24–42, GSE120221), after informed consent and institutional ethics approval.

For scRNA-seq, freshly resected bone and marrow tissues were stored in tissue-preservation solution (Miltenyi Biotec) until processing. Samples were washed with PBS, cut into small pieces, and digested at 37 °C for 50 min with Collagenase II (150 U/mL), Collagenase IV (2 mg/mL), Dispase II (1.2 U/mL), and DNase I (50 U/mL). The digests were filtered through a 70 µm cell strainer and centrifuged at 300 × *g* for 5 min. After red-blood-cell lysis (Miltenyi Biotec), cells were washed in PBS containing 0.04% BSA, passed through a 35 µm filter, and loaded onto the 10x Genomics Chromium Controller for single-cell capture using the Single Cell 3′ v3 reagent kit. Libraries were sequenced on an Illumina 150 bp paired-end run, and reads were aligned to the human reference genome (GRCh38) with CellRanger v3.1.0 to generate the single-cell barcode matrix. Cell types were defined from their gene-expression profiles using established marker genes for bone and immune cells (see Supporting Information). All downstream analyses, including Seurat-based integration and donor-level batch correction, as well as differential-expression and GSEA analyses, were performed in R (RStudio).

### Gene Ontology (GO) and KEGG pathway enrichment analysis

Differential expression analysis was carried out on donor-level pseudobulk profiles generated from the scRNA-seq data. For each clinical group (pre-menopausal, post-menopausal non-osteoporotic, and post-menopausal osteoporotic) and for each annotated cell type, we identified differentially expressed genes (DEGs) using a donor-adjusted one-versus-rest model implemented in the voom/limma pipeline. Genes with false discovery rate (FDR) < 0.05 and |log₂ fold change| > 0.25 were considered significant. Significant up- and down-regulated DEGs from each cell type were then used as input for Gene Ontology (GO) Biological Process and Kyoto Encyclopedia of Genes and Genomes (KEGG) pathway enrichment analyses using the clusterProfiler R package (v4.0). Enrichment results were expressed as adjusted *P* values (Benjamini–Hochberg correction).

### Statistical analysis

Data represent at least three independent experiments performed in triplicate unless stated otherwise. Statistical analysis was conducted using Prism 7.0 (GraphPad), with specific methods detailed in the figure legends. *P* values < 0.05 were considered significant, and error bars represent standard deviation (SD).

## Supplementary information

Supplementary Figures

Cell Annotation

## Data Availability

The data are available from the corresponding author on reasonable request.
