# Supplementary material for: Estradiol regulates osteoclast sialylation via ST3Gal1 in postmenopausal osteoporosis
Source: Bone Res. 2026 Feb 12;14:22. doi: 10.1038/s41413-025-00498-x (PMC12901315; doi:10.1038/s41413-025-00498-x)
Supplement: Supplementary file 1 — Supplementary Figures [file 41413_2025_498_MOESM1_ESM.docx]

**SUPPLEMENTARY MATERIALS**

**Supplemental Figure S1**


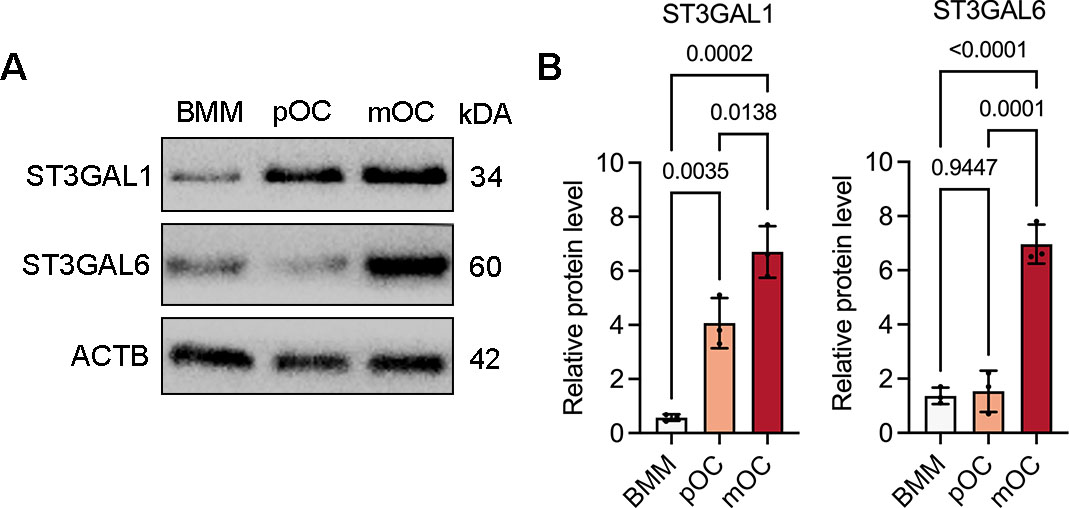


**Figure S1. Expression levels of ST3GAL1 and ST3GAL6 in different osteoclast differentiation stages**

**(A)** Representative western blots showing protein levels of ST3GAL1 (34 kDa) and ST3GAL6 (60 kDa) in bone marrow-derived macrophages (BMM), pre-osteoclasts (pOC), and mature osteoclasts (mOC). ACTB (β-actin, 42 kDa) is shown as a loading control.

**(B)** Quantification of relative protein expression levels of ST3GAL1 and ST3GAL6 normalized to ACTB in BMM, pOC, and mOC. Data are presented as mean ± SD (n=3). Statistical significance was determined by one-way ANOVA followed by Tukey’s post-hoc test. P-values are indicated for significant differences between groups.

**Supplemental Figure S2**


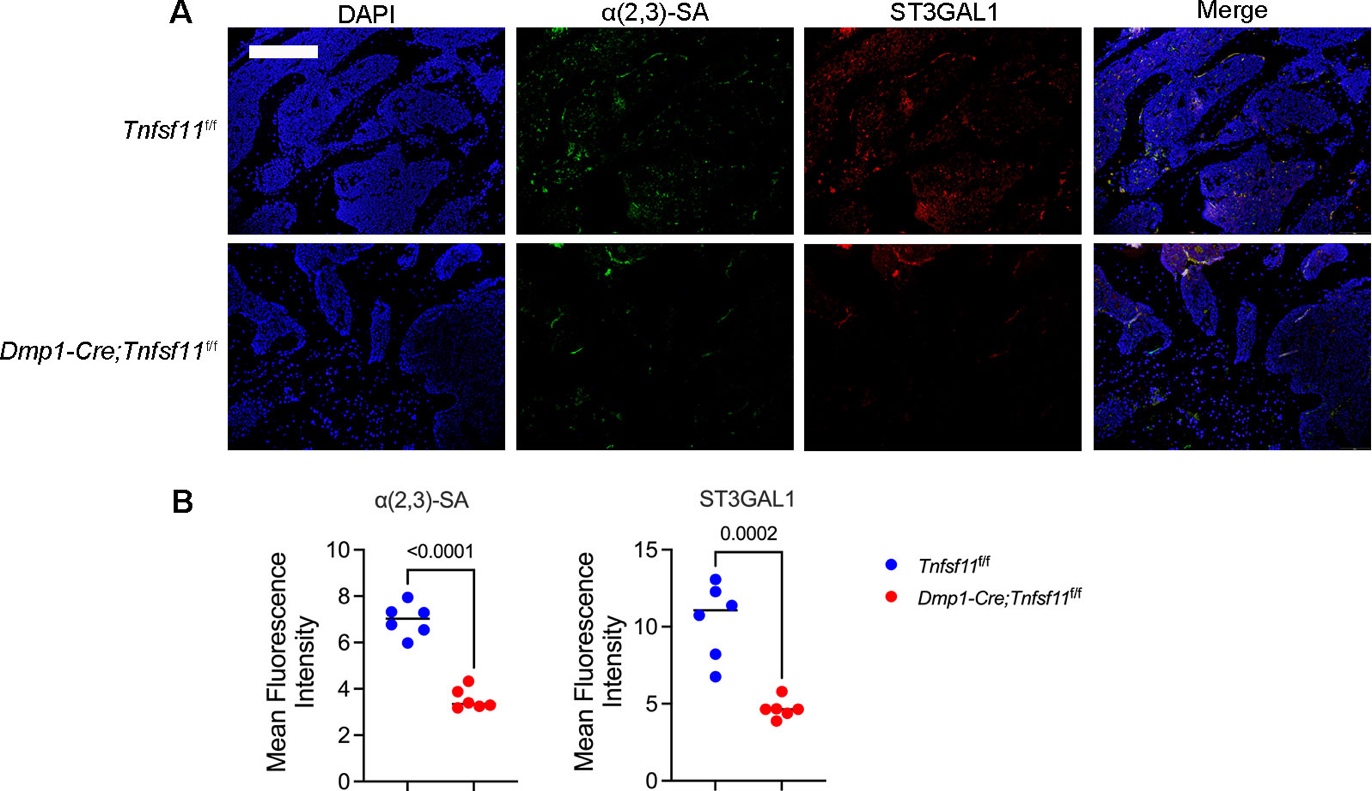


**Figure S2. Decreased expression of α(2,3)-sialic acid and ST3GAL1 in bone sections from Tnfsf11 conditional knockout mice**

**(A)** Immunofluorescence staining of α(2,3)-sialic acid (green) and ST3GAL1 (red) in bone sections from Tnfsf11^f/f^ (control) and Dmp1-Cre; Tnfsf11^f/f^ (conditional knockout) mice. Nuclei were counterstained with DAPI (blue). Scale bar = 100 µm.

**(B)** Quantification of mean fluorescence intensity for α(2,3)-sialic acid and ST3GAL1 from images shown in panel (A). Data are presented as mean ± SD (n=6). Statistical significance was determined using an unpaired two-tailed t-test. P-values are indicated for significant differences between groups.

**Supplemental Figure S3**


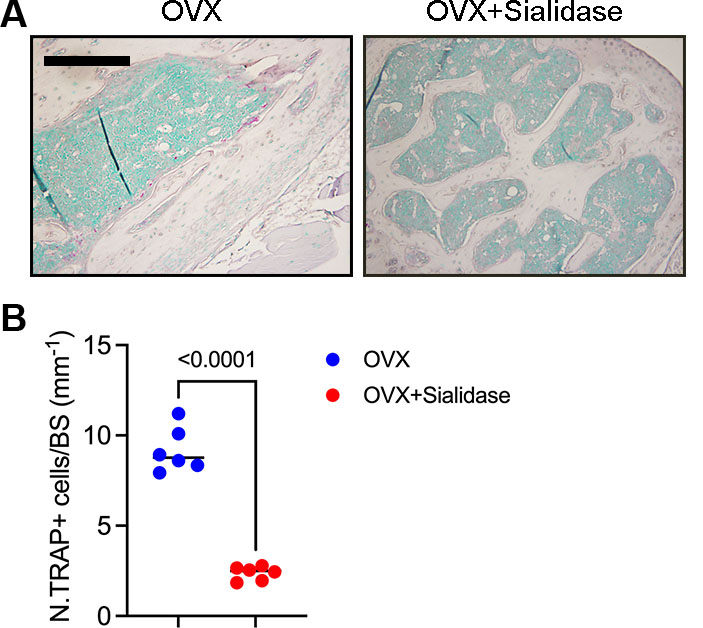


**Figure S3. Sialidase treatment reduces osteoclast numbers in ovariectomized (OVX) mice**

**(A)** Representative histological images of bone sections from OVX mice treated with or without sialidase, showing TRAP staining for osteoclasts. Scale bar = 200 µm.

**(B)** Quantification of TRAP-positive osteoclasts per bone surface (N.TRAP+ cells/BS, mm⁻¹) in bone sections from OVX and OVX + sialidase-treated mice. Data are presented as mean ± SD (n=6). Statistical significance was determined using an unpaired two-tailed t-test. P-values are indicated for significant differences between groups.


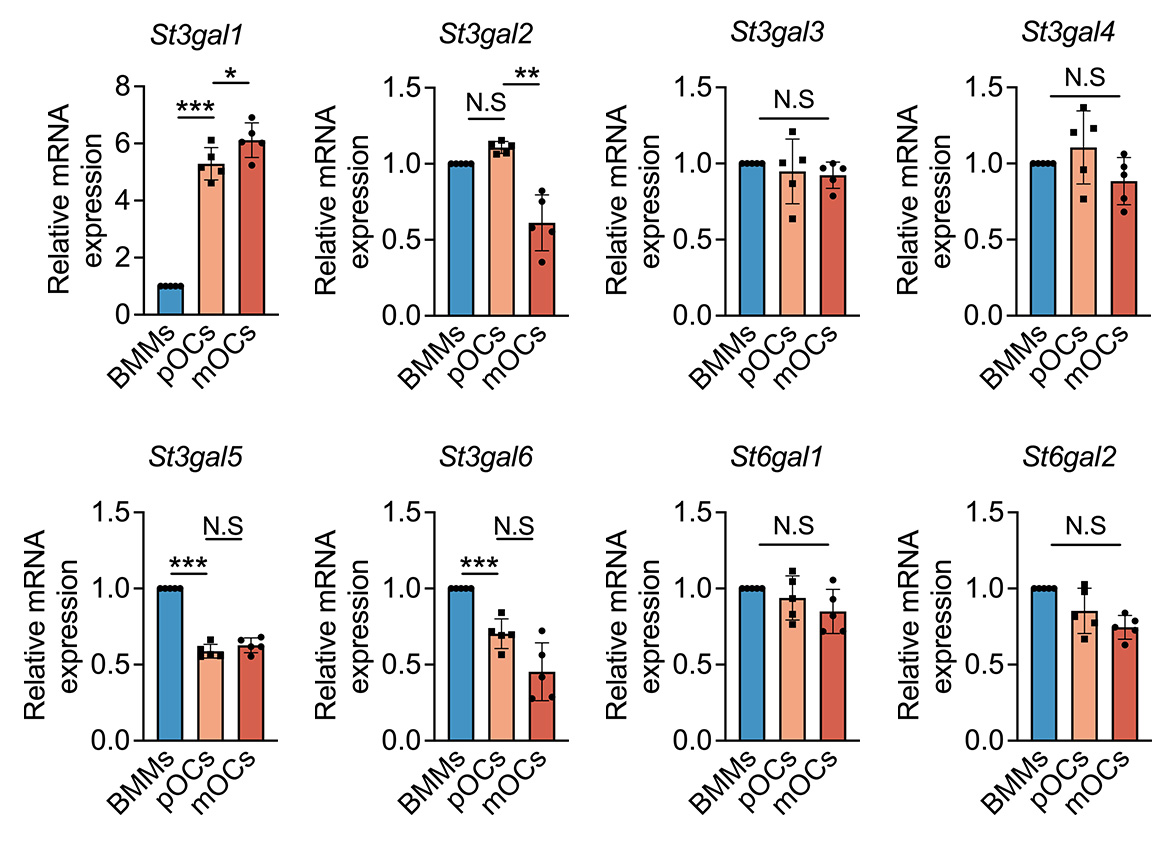


**Figure S4.** Quantitative PCR (qPCR) analysis of St3gal and St6gal family members across bone marrow macrophages (BMMs), pre-osteoclasts (pOCs), and mature osteoclasts (mOCs). Data are presented as mean ± SD; statistical significance was determined by one-way ANOVA, with **p* < 0.05, ***p* < 0.01, ****p* < 0.001, N.S. = not significant.


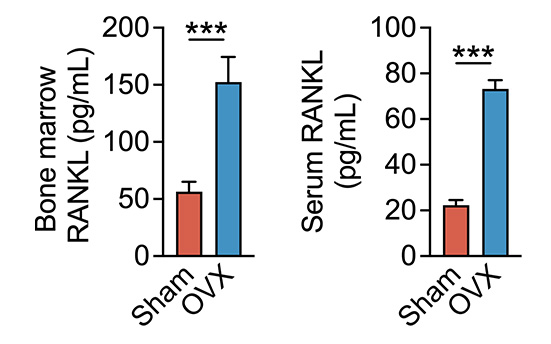


**Figure S5**. ELISA of RANKL in serum and bone marrow from sham and OVX mice.

**Figure S6**. Feature gene expression of ACP5, CTSK, and ST3GAL1-6, ST6GAL1 in BMMs, pOCs, and mOCs from mice.

**Figure S7**. Pseudotime trajectory analysis of bone marrow-derived cells from mice, showing the dynamic expression of St3gal1-4 during osteoclast differentiation. The plot illustrates the expression trends of these genes alongside canonical osteoclast markers Acp5 and Ctsk.


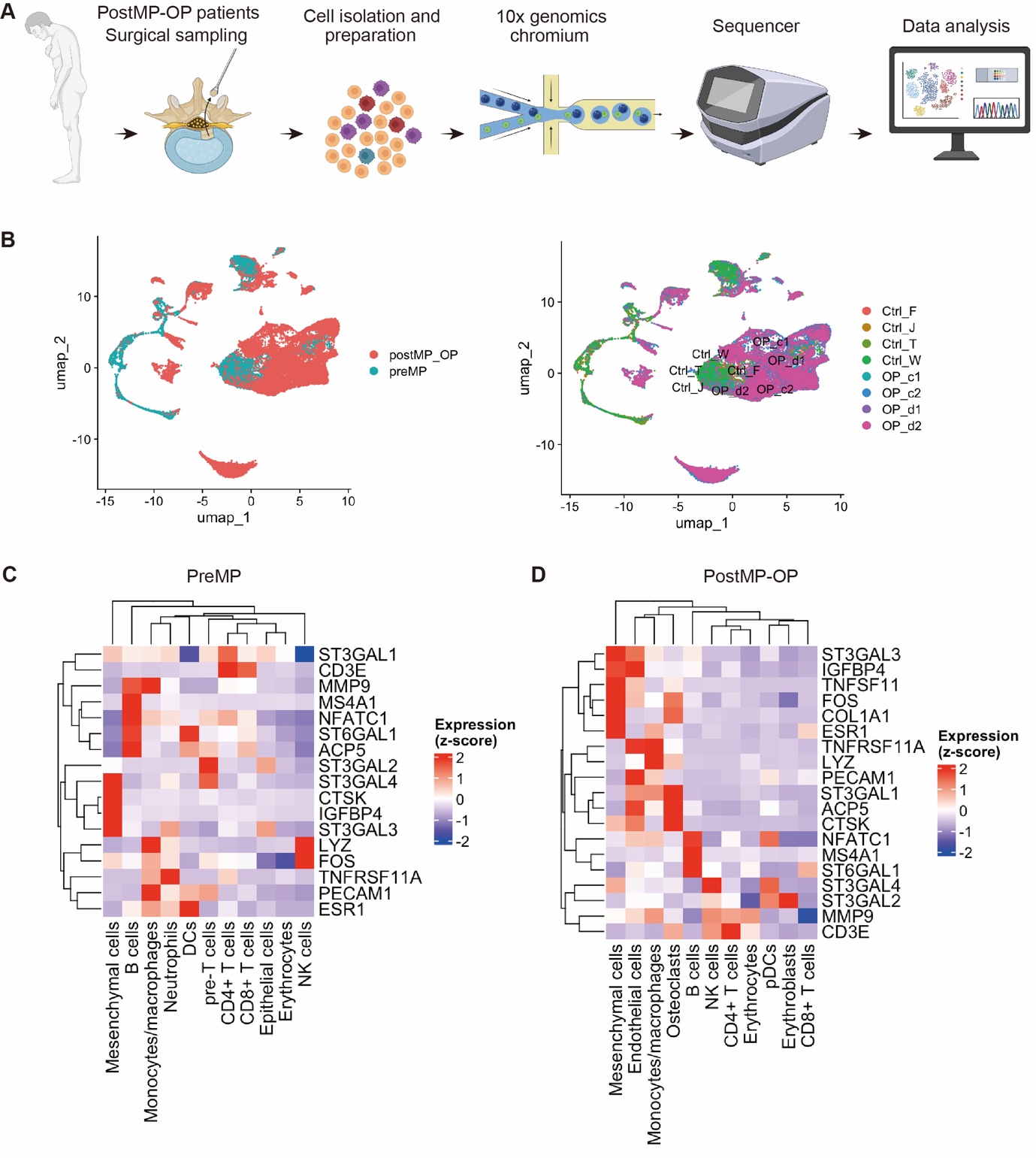


**Figure S8**. (A) Schematic workflow: surgical collection of bone samples from postmenopausal osteoporotic (postMP-OP) patients, single-cell dissociation, 10x Genomics Chromium capture and sequencing, followed by bioinformatic analysis. (B) UMAP visualization of all single cells coloured by clinical group (left: pre-menopausal [preMP] vs postMP-OP) and by individual donor (right). (C–D) Heatmaps showing z-scored average expression of selected genes involved in sialylation, osteoclast activity and estrogen signalling across annotated cell types in (C) preMP and (D) postMP-OP samples.


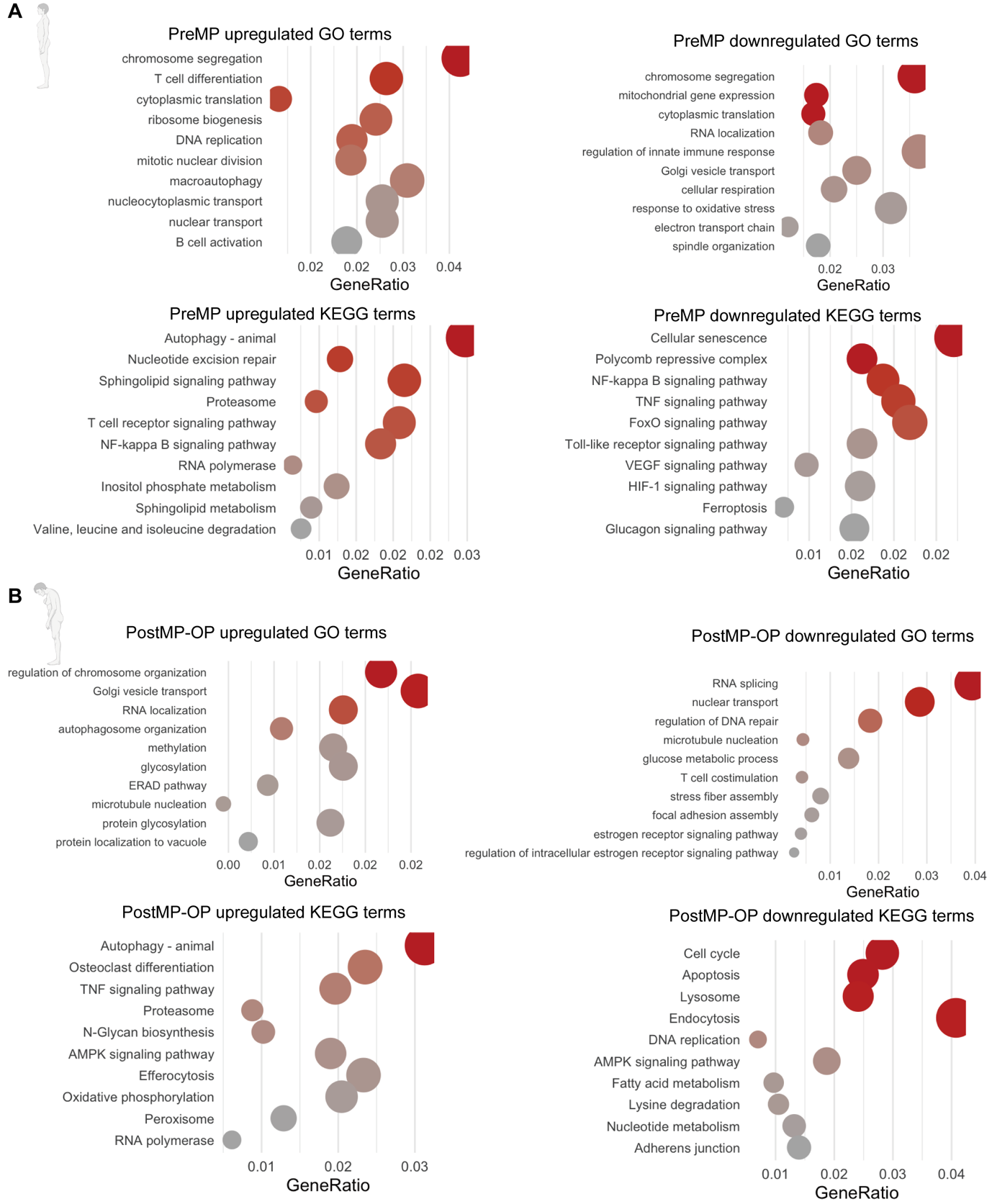


**Figure S9.** Gene Ontology (GO) and KEGG pathway enrichment of differentially expressed genes (DEGs) in pre- and post-menopausal bone marrow samples. (A) Bubble plots showing significantly enriched GO biological processes (top) and KEGG pathways (bottom) for genes up-regulated (left) or down-regulated (right) in pre-menopausal (PreMP) samples relative to post-menopausal osteoporotic (PostMP-OP) samples. (B) Corresponding GO (top) and KEGG (bottom) enrichment results for genes up- or down-regulated in PostMP-OP samples relative to PreMP. Bubble size reflects the GeneRatio (proportion of input genes in each term) and colour represents adjusted p-value.
