# Supplementary material for: Estradiol regulates osteoclast sialylation via ST3Gal1 in postmenopausal osteoporosis
Source: Bone Res. 2026 Feb 12;14:22. doi: 10.1038/s41413-025-00498-x (PMC12901315; doi:10.1038/s41413-025-00498-x)
Supplement: Supplementary file 2 — Cell Annotation [file 41413_2025_498_MOESM2_ESM.docx]

Each cell type was defined based on its unique gene expression profile, using marker genes commonly used in bone and immune cell studies:

• Osteoclasts: High expression of CTSK, ACP5 (TRAP), and NFATC1

• Osteoblasts: Express SP7 (Osterix), ALPL, BGLAP (Osteocalcin)

• Bone marrow stromal cells (BMSCs): Express PDGFRA, CD90 (THY1), and LEPR

• Monocytes/Macrophages: Defined by CD14, CD68, and ITGAM (CD11b)

• Macrophages: Express CD68, ITGAM (CD11b), and IL1B

• Neutrophils: Express CXCR2, FCGR3B, and S100A9

• Myeloid Cells: Express S100A8, CD33, and CSF1R

• B cells: Express CD19, CD20 (MS4A1), and CD79A

• CD4 T cells: Express CD3E, CD4, and CCR7

• CD8 T cells: Express CD3E, CD8A, and GZMA

• Exhausted T cells: Express CD3E, PDCD1 (PD-1), and LAG3

• Dendritic cells: Express CD11C (ITGAX), HLA-DR, and CD1C

• NK cells: Express NCAM1 (CD56), KLRD1 (CD94), and NKG2D (KLRK1)

• Fibroblasts: Express COL1A1, VIM (Vimentin), and DCN (Decorin)

• Endothelial Cells: Express IER3, CDH5 (VE-Cadherin), and PECAM1 (CD31)
